# Structural Basis for the Inhibition of *Helicobacter pylori* α-Carbonic Anhydrase by Sulfonamides

**DOI:** 10.1371/journal.pone.0127149

**Published:** 2015-05-26

**Authors:** Joyanta K. Modakh, Yu C. Liu, Mayra A. Machuca, Claudiu T. Supuran, Anna Roujeinikova

**Affiliations:** 1 Department of Microbiology, Faculty of Biomedical and Psychological Sciences, Monash University, Clayton, Victoria, Australia; 2 Laboratorio di Chimica Bioinorganica, Polo Scientifico, Università degli Studi di Firenze, Via della Lastruccia 3, Sesto Fiorentino (Florence) Italy; 3 Neurofarba Department, Sezione di Scienze Farmaceutiche, Università degli Studi di Firenze, Via U. Schiff 6, Sesto Fiorentino (Firenze), Italy; 4 Department of Biochemistry and Molecular Biology, Faculty of Biomedical and Psychological Sciences, Monash University, Clayton, Victoria, Australia; Institut Pasteur Paris, FRANCE

## Abstract

Periplasmic α-carbonic anhydrase of *Helicobacter pylori* (HpαCA), an oncogenic bacterium in the human stomach, is essential for its acclimation to low pH. It catalyses the conversion of carbon dioxide to bicarbonate using Zn(II) as the cofactor. In *H*. *pylori*, *Neisseria* spp., *Brucella suis* and *Streptococcus pneumoniae* this enzyme is the target for sulfonamide antibacterial agents. We present structural analysis correlated with inhibition data, on the complexes of HpαCA with two pharmacological inhibitors of human carbonic anhydrases, acetazolamide and methazolamide. This analysis reveals that two sulfonamide oxygen atoms of the inhibitors are positioned proximal to the putative location of the oxygens of the CO_2_ substrate in the Michaelis complex, whilst the zinc-coordinating sulfonamide nitrogen occupies the position of the catalytic water molecule. The structures are consistent with acetazolamide acting as site-directed, nanomolar inhibitors of the enzyme by mimicking its reaction transition state. Additionally, inhibitor binding provides insights into the channel for substrate entry and product exit. This analysis has implications for the structure-based design of inhibitors of bacterial carbonic anhydrases.

## Introduction

*Helicobacter pylori* is a pathogenic bacterium that colonises the stomach of approximately 50% of the human population [1]. *H*. *pylori* infections are associated with severe gastroduodenal diseases such as gastritis, peptic ulcers and gastric cancers [2–5]. Current *H*. *pylori* eradication therapies rely on the simultaneous use of two or more broad-spectrum antibiotics (commonly amoxicillin and clarithromycin) [6] and a proton pump inhibitor [7]. However, recent reports show that this combination has lost efficacy, with an eradication rate ranging from 71% in the United States to 60% in Western Europe [8–10]—well below the expected rate of 80% for first line therapy [11]. Therefore, there is a growing need to identify and develop a more effective alternative to traditional therapies.

Bacterial carbonic anhydrases (CAs, EC 4.2.1.1), metalloenzymes that catalyse the hydration of carbon dioxide to bicarbonate and hydrogen ions, are emerging as new potential drug candidates due to their role in the survival, invasion and pathogenicity of bacteria [12, 13]. *H*. *pylori* has two different CAs, α-class and β-class (HpαCA and HpβCA) [14]. Joint activities of α- and β-CAs and urease are required to produce NH_3_/NH_4_^+^ and CO_2_/HCO_3_^-^ couples that maintain *H*. *pylori* periplasmic and cytoplasmic pH close to neutral in the highly acidic medium of the stomach, thus allowing both survival and growth in the gastric niche [15, 16]. HpαCA and HpβCA are highly inhibited by many primary sulfonamides RSO_2_NH_2_, including the clinical drugs acetazolamide (AAZ), ethoxzolamide, methazolamide (MZA), topiramate and sulpiride [17, 18]. Furthermore, certain CA inhibitors, such as acetazolamide and methazolamide, were shown to inhibit the *H*. *pylori* growth in cell cultures [19]. In addition, previous studies have shown that treating *H*. *pylori* with CA inhibitors drastically reduces the ability of the bacteria to survive within an acid environment, suggesting that CAs are essential for colonisation of the stomach and duodenum [20, 21]. Apart from *H*. *pylori*, several other Gram negative and Gram positive bacterial species display susceptibility to CA inhibitors, including *Neisseria* spp. [22], *Brucella suis* [23] and *Streptococcus pneumoniae* [24], which highlights the potential of the sulfonamide CA inhibitors as lead compounds for developing novel anti-infective agents.

Evidence that *H*. *pylori* CA inhibitors may be effective *in vivo* comes from pilot studies of the treatment of peptic ulcer disease with AAZ. Treatment for 30 days achieved 96–97% of gastric and duodenal ulcer healing [25, 26]. Whilst the healing effect was partly attributable to inhibition of human CA activity in the parietal cells of the patients which caused suppression of basal secretion of gastric acid [27], it has become apparent that AAZ treatment also likely resulted in eradication of *H*. *pylori*. Indeed, AAZ was shown to be sufficiently effective not only in ulcer healing but also in prevention of ulcer recurrence. Two years after antiulcer therapy was discontinued, the recurrence rate in patients treated with AAZ (6%) was significantly lower than that with classical antacid drugs (30–60%) [25, 28] and close to that achieved by the triple eradication therapy [28]. Taken together, these clinical results suggested that, apart from antiacid action, AAZ had an inhibitory effect on *H*. *pylori*, a causative pathogen for peptide ulcer disease.

**Fig 1:**
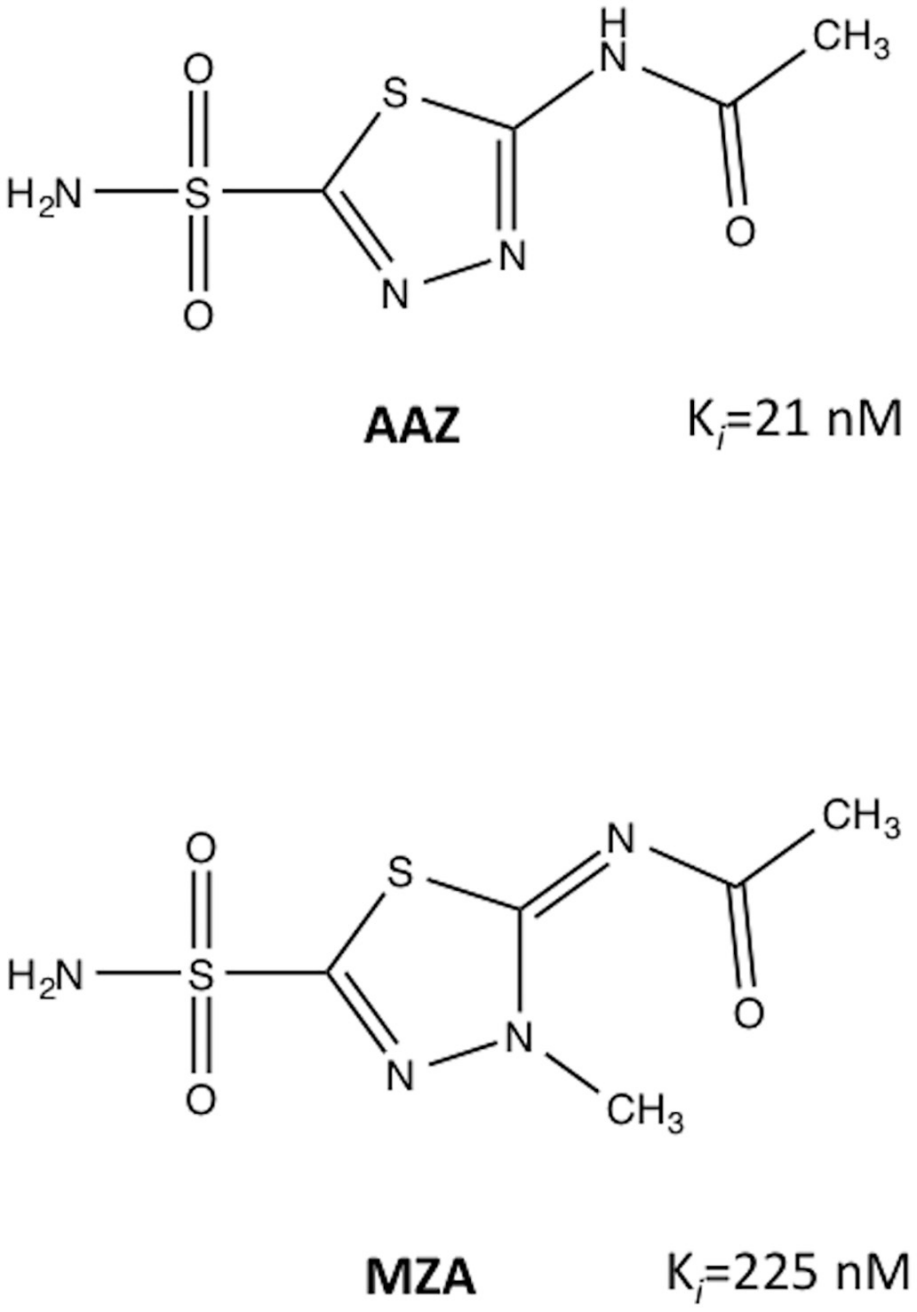
Structures of sulfonamides discussed in this study. (**AAZ**) acetazolamide, *N*-(5-sulfamoyl-1,3,4-thiadiazol-2-yl)acetamide; (**MZA**) methazolamide, (*E*)-*N*-(3-methyl-5-sulfamoyl-1,3,4-thiadiazol-2(3*H*)-ylidene)acetamide. The values for the inhibitory constants are as reported previously [18].

Here, we report structural analyses correlated with inhibition data, on complexes of HpαCA with AAZ and MZA (Fig 1). This study has allowed us to address the molecular details of catalysis of this enzyme and led to the identification of the protein elements that play a role in inhibitor recognition.

## Materials and Methods

### Protein purification, crystallisation and data collection

HpαCA is a homo-dimer in solution with a subunit mass of approximately 27 kDa and was prepared as described [29]. AAZ and MZA were purchased from Sigma-Aldrich. Crystals of the complexes with AAZ and MZA were obtained by mixing 8 mg/ml protein with 1 mM AAZ or MZA, 1 mM ZnCl_2_, 12% (w/v) PEG 1.5K and 100 mM di-basic ammonium citrate and suspending 2-μl drops over a well solution containing 24% (w/v) PEG 1.5K, 200mM di-basic ammonium citrate. Diffraction data were collected at cryogenic temperatures using the MX1 and MX2 beamlines of the Australian Synchrotron. All data were processed and scaled using *iMOSFLM* [30] and SCALA [31] from the CCP4 software suite [32]. Data collection statistics are summarised in Table 1. The crystals of all complexes were isomorphous and belonged to space group *P*2_1_ with the β value close to 90°, the condition under which pseudo-merohedral twinning can occur. Analysis of the data using PHENIX Xtriage [33] detected pseudo-merohedral twinning with the twin law (h, -k, -l).

**Table 1:** X-ray data collection statistics.

Complex	AAZ	MZA
Space group	*P*2_1_	*P*2_1_
Cell dimension (*a*, *b*, *c* (Å), *β* (°)	41.8, 133.6, 166.5, 90.2	42.5, 133.7, 166.6, 90.1
Observed reflections	411397	351477
Unique reflections	119874	93672
Resolution range (Å)	33.4 - 2.0 (2.1 - 2.0)	30.0 - 2.2 (2.3 - 2.2)
R_merge_ ^1^	0.096 (0.250)	0.078 (0.220)
Average I/σ(I)	7.7 (3.6)	11.0 (5.3)
Completeness (%)	97.6 (92.6)	98.2 (97.7)
Redundancy	3.4 (3.2)	3.8 (3.8)

^1^
$$Rmerge=\frac{\left(\sum_{h}\sum_{i}\left|I_{hi}-\langle I_{h}\rangle \right|\right)}{\sum_{h}\sum_{i}\left|I_{hi}\right|}\text{,}$$
where ***I***_*hi*_ is the intensity of the *i*th observation of reflection *h*.

### Structure determination and analysis

The crystal structure of the HpαCA complex with AAZ was determined using molecular replacement (PHASER) [34] with the structure of αCA from *Sulfurihydrogenibium yellowstonense YO3AOP1* (SspCA, PDB ID 4G7A; [35]) as a search model. Eight copies of the search model, corresponding to four dimers, were found in the asymmetric unit. Model building and refinement were carried out using the programs COOT [36] and PHENIX [37], with the twin law (h, -k, -l) and non crystallographic symmetry (NCS) restraints. The Fourier difference maps clearly revealed density for one Zn ion and one AAZ molecule in each subunit. The average B factors for the Zn ions and AAZ molecule in the final refined model (22 and 23 Å^2^, respectively) were close to that of the surrounding protein atoms, indicating that both Zn and the inhibitor molecule are bound with an occupancy close to 1.

The structure of the HpαCA complex with MZA was solved by molecular replacement using the protein coordinates of the dimer of the HpαCA/AAZ complex as a search model. Analysis of the stereochemical quality of the model was accomplished using MOLPROBITY [38]. The refinement statistics are summarised in Table 2. Structure figures were prepared using PYMOL [39]. The sequence alignment figure was produced using ESPript (http://espript.ibcp.fr) [40]. The coordinates and structure factors for the HpαCA complexes with AAZ and MZA have been deposited in the Protein Data Bank (www.rcsb.org) under accession codes 4YGF and 4YHA, respectively.

**Table 2:** Properties of the final models.

	HpαCA-AAZ	HpαCA-MZA
Resolution range (Å)	30.0 - 2.0	30.0 - 2.2
No. of reflections	119722	93642
Residues/atoms/waters	1795/ 14731/1092	1773/ 14546/ 665
R/R_free_	0.18/0.21	0.19/0.23
Average B (protein) (Å^2^)	29	39
Average B (water) (Å^2^)	26	32
Average B (Zn ions) (Å^2^)	22	45
Average B (inhibitors) (Å^2^)	23	35
Bond-length deviation from ideality (Å)	0.01	0.01
Bond-angle deviation from ideality (°)	1.5	1.5
Ramachandran space (%)		
Favored	96	95
Allowed	4	5
Outliers	0	0

## Results and Discussion

### Overall structure of HpαCA and comparison to other bacterial αCAs

**Fig 2:**
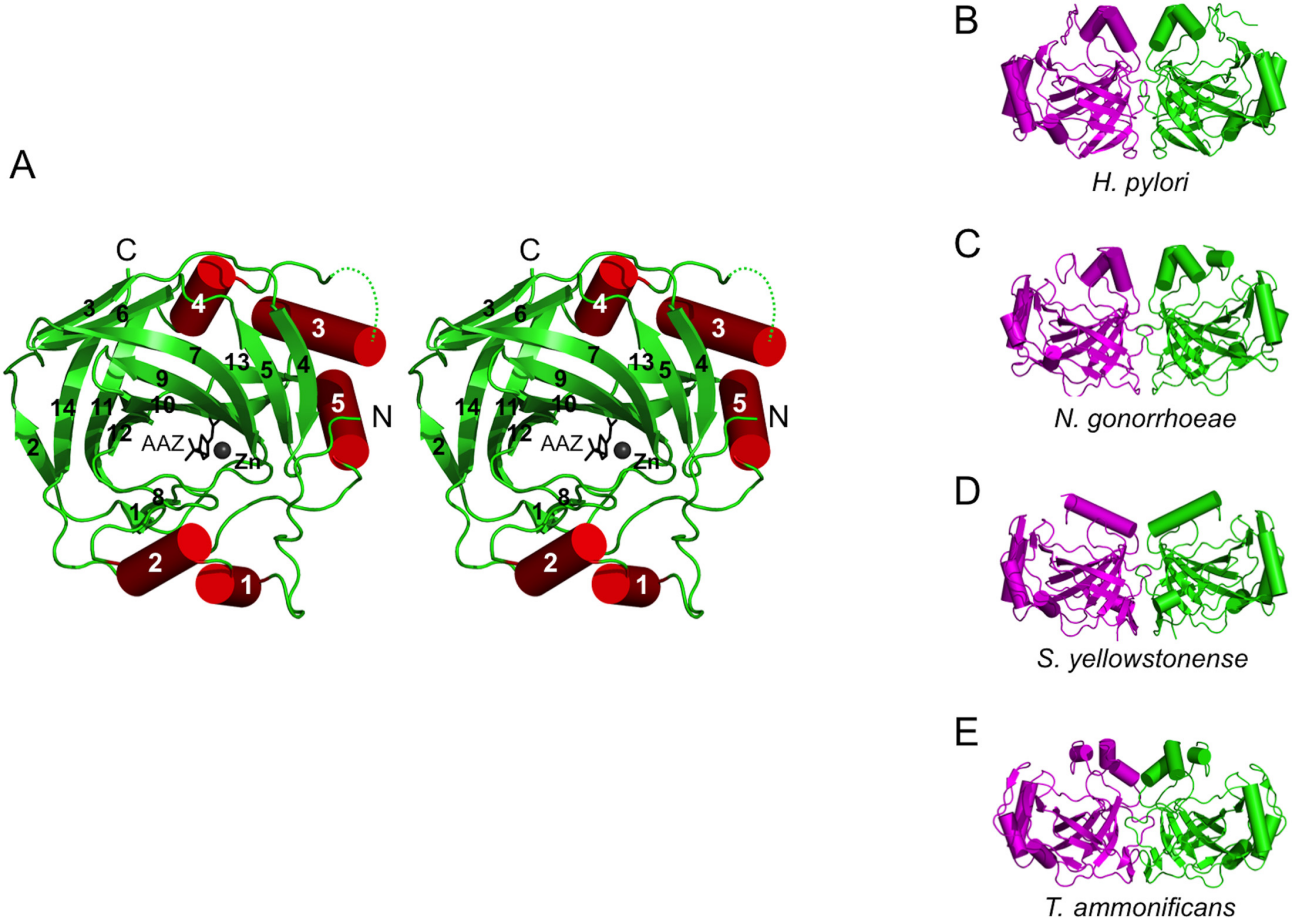
The crystal structure of HpαCA and comparison with other bacterial CAs. A: Stereo diagram of the structure of the HpαCA monomer. Each element of the secondary structure is labeled. The zinc ion and AAZ molecule are shown in ball and in stick representation, respectively, to indicate the location of the active site. B, C, D, E: Dimers observed in the crystal structures of HpαCA, NgCA, SspCA and TaCA, respectively.

The HpαCA subunit consists of a single polypeptide chain of 228 residues and comprises a total of 5 α-helices and 14 β-strands, which fold into a compact globular domain approximately 43 × 48 × 50 Å^3^ in size (Fig 2A). The β strands and α-helices are arranged in the topological order ααββββββββββααβββαβ. The structure reveals a fold characteristic of other αCAs of the known structure [41] which contains a central 10-stranded, twisted β-sheet flanked by helices α1 and α2 on one side and α3–α5 on the other. In a comparison of HpαCA against the structures in the RCSB Protein Data Bank [42] that have been described in the literature, using the protein structure comparison service Fold at European Bioinformatics Institute (http://www.ebi.ac.uk/msd-srv/ssm) [43], significant similarities were found with other bacterial αCAs. HpαCA has the closest structural similarity to the homologous enzymes from *Thermovibrio ammonificans* (TaCA, PDB ID code 4C3T [44]), *Neisseria gonorrhoeae* (NgCA, PDB ID code 3R9G [45]) and *S*. *yellowstonense* (SspCA) (rms deviation of 1.2 Å, 1.5 Å and 1.3 Å for the superimposition of 205, 214 and 205 C_α_ atoms, showing 36%, 38% and 39% sequence identity over equivalent positions, respectively). Inspection of the superimpositions showed that structural similarity extends over the entire fold and includes all the secondary structure elements.

We have previously shown that when subjected to gel-filtration, HpαCA eluted as a single peak with an apparent molecular weight of approximately 50 kDa, indicating that HpαCA forms a dimer in solution [29], in line with previous reports of the dimeric state of αCAs from *S*. *yellowstonense* [35] and *N*. *gonorrhoeae* [45]. Analysis of the packing of the eight HpαCA monomers in the asymmetric unit identified an obvious dimer with 2-fold symmetry and approximate dimensions of 43 × 50 × 84 Å (Fig 2B). Eleven percent (1268 Å^2^) of the subunit accessible surface area is buried upon dimerisation, which falls within the range found for other dimeric proteins [46]. In line with this, analysis of probable assemblies in the crystal using the PDBe PISA server (http://www.ebi.ac.uk/msd-srv/prot_int/cgi-bin/piserver) also suggested that HpαCA likely exists as a stable dimer in solution. The HpαCA dimer is very similar to that observed in the crystals of NgCA, SspCA and TaCA (Fig 2C–2E), providing further evidence in support of the hypothesis that, unlike mammalian αCAs, which are predominantly monomeric, bacterial αCAs are dimeric. An interesting variation in this series is that two TaCA dimers assemble into a tetramer by means of intra-subunit disulfide bonds, which is thought to contribute to the TaCA’s thermostability [46].

**Fig 3:**
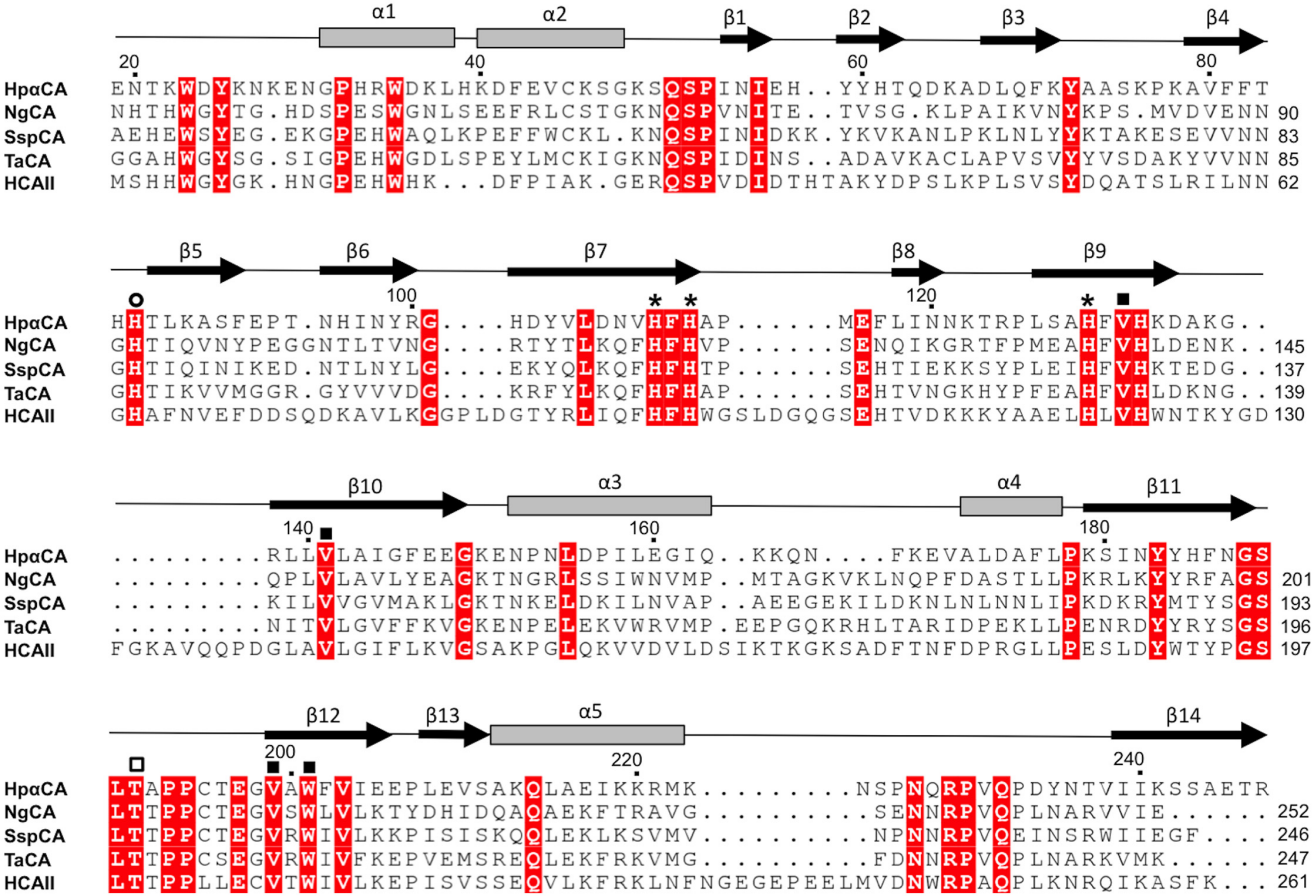
Structure-based sequence alignment of HpαCA, NgCA, SspCA, TaCA and HCAII. The elements of the secondary structure and the sequence numbering for HpαCA are shown above the alignment. Conserved residues are highlighted in red. The conserved histidine residues coordinating the zinc ion are marked by an asterisk. The proton shuttle residue of HCAII (His64) is marked with an open circle. The corresponding residue in HpαCA is His85. The conserved residues that form a hydrophobic pocket in the active site are labeled with filled squares. The location of the catalytically important HCAII residue Thr199 which forms a hydrogen bond to the zinc-bound water (Wat263, Fig 5), thereby orienting its two lone electron pairs toward the two neighbouring water molecules (Wat318 and Wat338) in the active site is shown by an open square. The corresponding residue in HpαCA is T191.

Further structural comparisons show that the HpαCA fold is very similar to mammalian αCAs, the closest homologue (28% sequence identity) being human carbonic anhydrase II (HCAII) [17]. Superimposition of the structures of HpαCA and HCAII (PDB ID code 3DVC [47]) reveals that 206 of 258 C_α_ atoms could be superimposed with an rmsd of 1.6 Å showing 32% identity over the aligned amino acid residues. Most of the secondary structure elements present in HCAII are retained in HpαCA. The structural differences between the two enzymes are mainly in the length of the surface loops (Fig 3): loops corresponding to β6-β7, β7-β8,β9-β10, α3-α4 and α5-β14 in HpαCA are longer in HCAII.

### Zinc and acetazolamide binding and localisation of the active site

A conserved key feature of the active site of αCAs is the zinc ion (cofactor) located in a cone-shaped cavity and coordinated tetrahedrally by three histidine residues [41]. Previous comparison of the sequence of HpαCA with that of HCAII highlighted the conservation of the residues involved in zinc coordination (His110, His112 and His129 (Fig 3)), binding of the substrate (Leu190) and proton shuttling (His85) [19]. Compared with HCAII these residues in HpαCA lie in equivalent positions on the side of the core β-sheet. They reside on strands β7 (His110, His112), β9 (His129) and on the loops β11-β12 (Leu190) and β4-β5 (His85) at the bottom of the long conical cavity, which clearly identifies it as the active site. In common with other bacterial αCAs, the entrance into this cavity in HpαCA is wider than in HCAII due to the shorter loop connecting strands β9 and β10.

**Fig 4:**
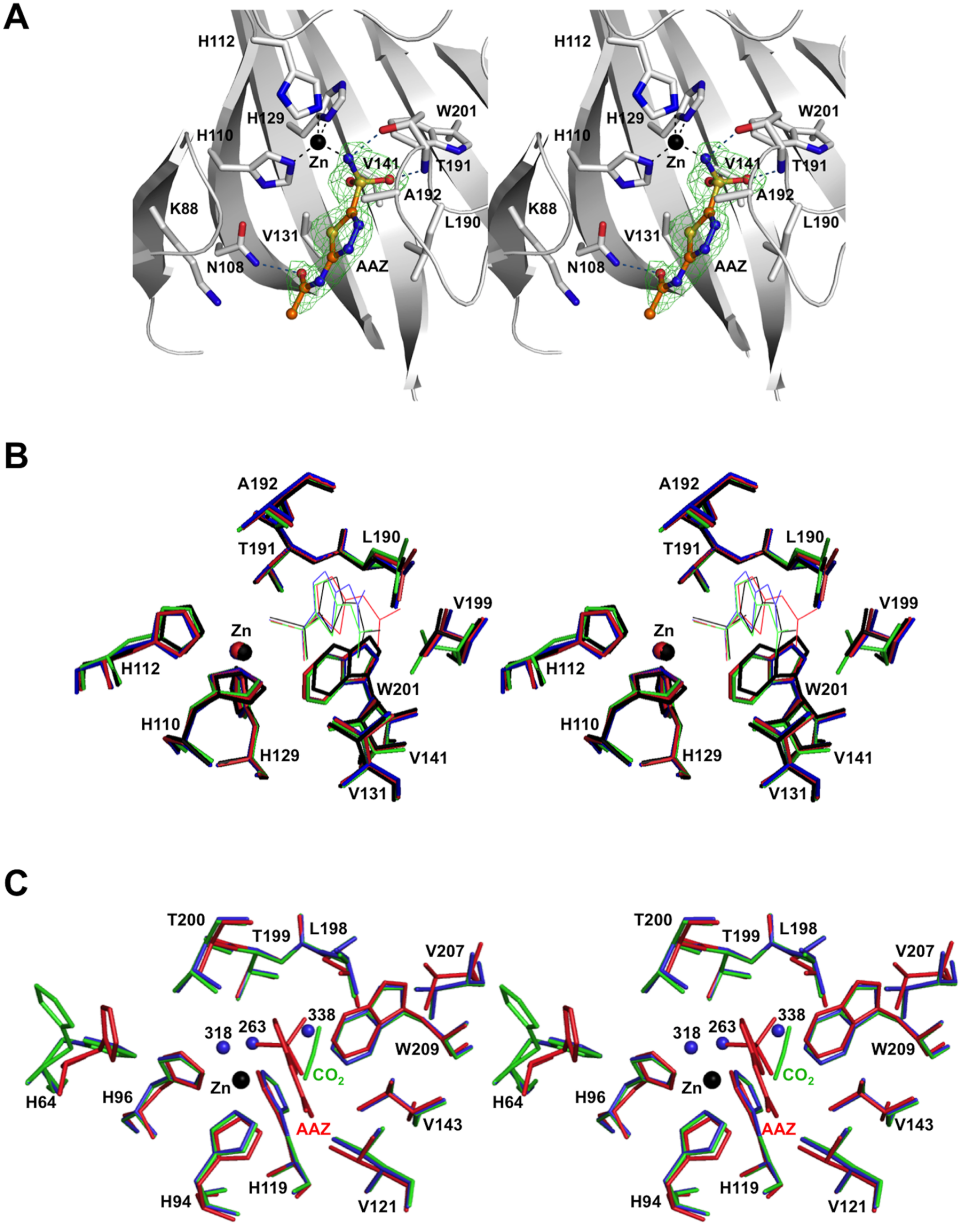
Stereo view of the AAZ-binding site in HpαCA and comparison to other αCAs. A: The (mFo-DFc) sigmaA-weighted [32] electron density for AAZ bound to HpαCA is shown in green. The map was calculated at 2.0-Å resolution and contoured at 3.0-σ level. The AAZ molecule is shown in all-atom ball-and-stick representation with carbon atoms coloured orange. Amino acid residues that interact with AAZ are shown in stick representation. The catalytic zinc ion that is coordinated tetrahedrally (black dash lines) is shown as a black sphere. Hydrogen bonds are shown as blue dashed lines. B: Stereo view of the inhibitor-binding sites of the superimposed HpαCA/AAZ (black), SspCA/AAZ (red), TaCA/AAZ (blue) and HCAII/AAZ (green) complexes. The diagram illustrates the remarkably similar mode of AAZ binding, particularly around the sulfonamide moiety which coordinates the zinc ion. Residues are labeled in HpαCA. C: Stereo view of the superposition of the active site regions of HpαCA (red) and HCAII (blue for the free enzyme, green for the complex with CO_2_). Residues and water molecules are labeled in HCAII. The “deep” water (Wat338), catalytic water (Wat263) and Wat318 observed in the active site of free HCAII are shown as blue spheres. The CO_2_ (substrate) observed in the crystal structure of the HCAII/CO_2_ complex is shown in green. The zinc ion (black sphere) is only shown in the free HCAII structure for clarity.

**Fig 5:**
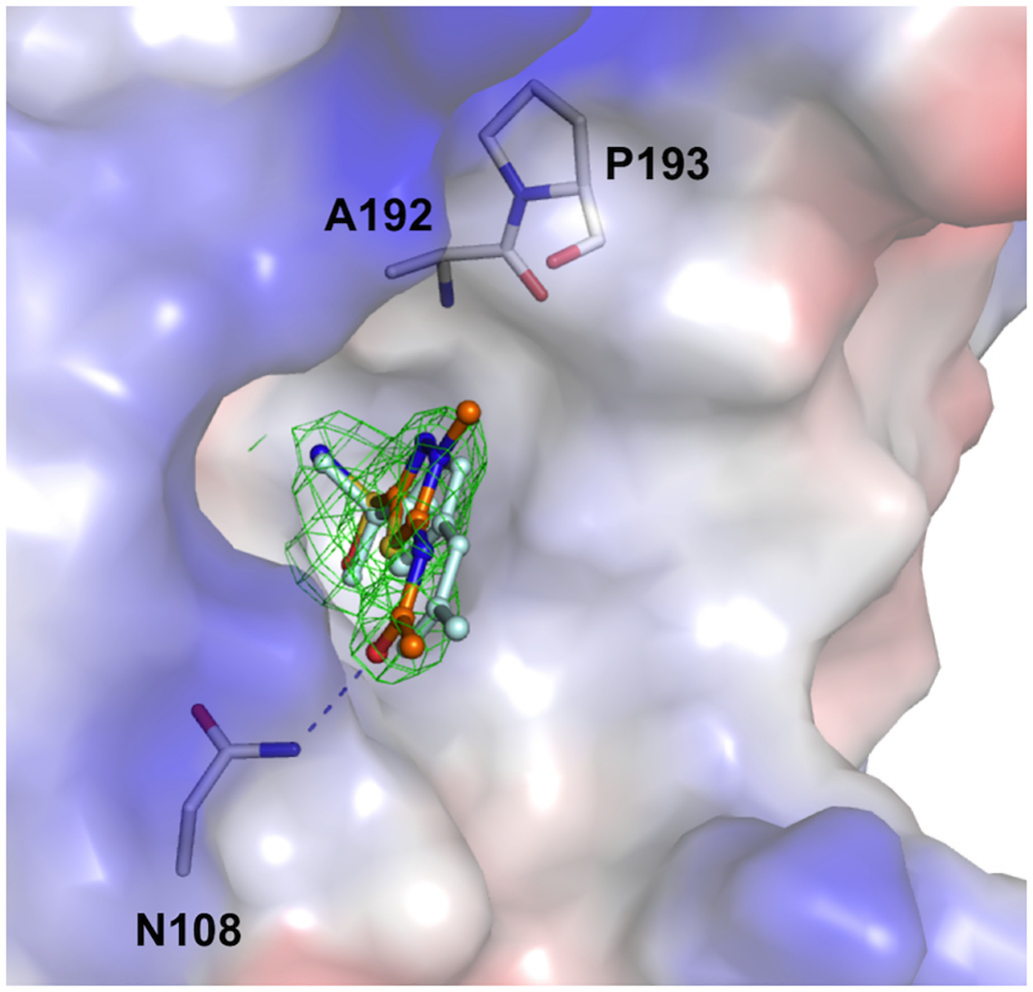
Structural comparison between the MZA and AAZ complexes of HpαCA. The MZA molecule is shown in all-atom representation with carbon atoms coloured in orange. The (mFo-DFc) sigmaA-weighted electron density for MZA is shown in green. The map was calculated at 2.2-Å resolution and contoured at 3.0-σ level. The AAZ molecule is shown in cyan. Protein surface is shown and coloured according to the electrostatic potential. The subtle rotation and shift of MZA with respect to AAZ does not break the hydrogen bond between the O3 atom of the carbonamide moiety and the side chain of Asn108. The weaker binding of MZA in comparison to AAZ is likely due to energetically unfavourable interaction between the additional aliphatic methyl group of MZA and partially negatively charged carbonyl oxygens of the main-chain peptides of Ala192 (C-O distance 4.3 Å) and Pro193 (C-O distance 3.5 Å).

The zinc ion has been identified in the structures of both AAZ and MZA complexes as a strong peak on the difference map at the position coordinated by His110, His112 and His129. Furthermore, difference-Fourier maps revealed clear and unambiguous density for AAZ and MZA in their respective complexes (Figs 4A and 5). In both structures, the N atom of the sulfonamide moiety of the inhibitor acts as the fourth ligand in the tetrahedral coordination sphere for the zinc ion. This atom is also within hydrogen bonding distance from the Oγ atom of Thr191 (2.6 Å) (Fig 4A). The O1 atom of the sulfonamide group forms a hydrogen bond with the main-chain amide of Thr191. The sulfonamide group of AAZ is further stabilised by van der Waals contacts with the side chains of Val141 and Trp201. The thiadiazole moiety of AAZ stacks against the hydrophobic side of the cone-shaped surface leading into the active site and makes van der Waals contacts with Val131, Leu190 and Ala192. The AAZ molecule is further stabilised through a hydrogen bond between the O3 atom of its acetamido group and the Nδ atom of Asn108 (3.1 Å) and van der Vaals contact with the side chain of Lys88. Structural superimpositions showed that the positions of the AAZ molecule and the zinc ion are very similar in all eight subunits in the asymmetric unit. Superposition of the structures of the HpαCA/AAZ, SspCA/AAZ, TaCA/AAZ and HCAII/AAZ complexes shows a very good overlap of the zinc-coordinating sulfonamide moieties and only subtle differences in the orientation of the AAZ molecule, consistent with the absolute conservation of the protein residues that are important for AAZ recognition (Fig 4B).

### Active site structure and an insight into the catalytic mechanism

Superposition of the HpαCA/AAZ complex with the structures of free and CO_2_-bound HCAII (PDB ID codes 2CBA and 2VVA [48]) reveals that many residues that line the walls of the AAZ binding pocket in HpαCA are conserved in HCAII, where they play an important role in catalysis (Fig 4C). Amino acid residue His85 of HpαCA corresponds to HCAII residue His64, which is the catalytic proton shuttle that undergoes transition between the inward and outward conformations and thus facilitates proton transfer between the active site and surrounding solvent in catalysis by HCAII [48]. As in other previously characterised bacterial αCAs [44, 45], the imidazole ring of His85 in the crystal structure of HpαCA is pointing away from the active site, most likely due to repulsion between the positively charged imidazole ring of the solvent-exposed His85 and zinc ion under the acidic conditions of the crystallisation mix. Amino acid residues Val131, Val141, Leu190 and Trp201 of HpαCA correspond to HCAII residues Val121, Val 143, Leu198 and Trp209, that form the hydrophobic pocket that is important for recognition and correct orientation of the CO_2_ molecule. Thr191 in HpαCA corresponds to HCAII residue Thr199 that forms a hydrogen bond to the zinc-bound catalytic water/hydroxide ion (Wat263, Fig 4C), thereby orienting its two lone electron pairs toward the two neighbouring water molecules (Wat318 and Wat338) that reside on potential substrate-binding sites in HCAII. Residue Thr200 in HCAII which forms part of the hydrogen-bonding network connected to the proton shuttle His64 in HCAII is not conserved in HpαCA; the latter has alanine in this position. However, there is no experimental evidence for the essential role of Thr200 in HCAII catalysis. The observation that the side chains of all residues that are implicated in the catalytic mechanism of HCAII are conserved in HpαCA and their positions and conformations are very close to those of respective residues in the human homologue strongly suggests that HpαCA is likely to follow the same reaction mechanism where CO_2_ is converted into bicarbonate HCO_3_^-^ *via* a nucleophilic attack on CO_2_ by the reactive zinc-bound hydroxide ion [41, 49].

In addition, examination of the superposition of the HpαCA/AAZ complex with the structures of free and substrate (CO_2_)-bound HCAII reveals that two sulfonamide oxygen atoms of AAZ are positioned close to the putative location of the oxygens of the CO_2_ substrate in the Michaelis complex (0.8 Å and 0.4 Å) (Fig 4C). Furthermore, in this superimposition the zinc-coordinating sulfonamide nitrogen occupies the position of the catalytic water/hydroxide ion Wat263. Thus, our analysis suggests that AAZ acts as a site-directed inhibitor by mimicking the catalytic transition state of the CO_2_ hydration reaction, which is in agreement with the tight binding reported for this inhibitor (K_*i*_ = 21 nM) [18]. Additionally, it can be deducted that the surface-exposed aliphatic amino acid residues Val131, Leu190 and Ala192 that form stabilising interactions with the thiadiazole and acetamido moieties of AAZ likely line the wall of the channel for substrate entry and product exit.

### Methazolamide binding

The chemical structure of MZA is very close to that of AAZ, with the main difference being the substitution with CH_3_ at position 3 of the thiadiazole ring (Fig 1). Superposition of the structures of the HpαCA complexes with AAZ and MZA (Fig 5) shows that the mode of binding of MZA is very close to that of AAZ except for the subtly different orientation of the thiadiazole ring: in the structure of the MZA complex the plane of this ring is tilted by 5° relative to the plane of the same ring in the superimposed AAZ complex, so that there is no steric clash between the methyl group and the protein atoms. The overall hydrogen bonding network stabilising MZA is identical to that of AAZ. Furthermore, the calculated values of the relative accessible surface area buried upon interaction of AAZ and MZA with protein are very close (42% and 40% respectively). Our structural analysis therefore suggests that, with all other structural factors being similar, the 10-fold difference in the inhibitory constant (K_*i*_ (MZA) = 225 nM) is likely explained by the energetically unfavourable interaction between the aliphatic methyl group of MZA and partially negatively charged main-chain carbonyl oxygens of Ala192 and Pro193 (Fig 5).

### Concluding Remarks

Here we presented analysis of the first crystal structure of αCA from the carcinogenic bacterium *H*. *pylori*, which allowed us to address the molecular details of catalysis of this enzyme. In addition, we revealed the structural basis of inhibition of HpαCA by AAZ and MZA. Both compounds are also inhibitors of human CAs and have been used clinically, originally as diuretics [50], and subsequently as antiglaucoma or antiulcer drugs [51, 52]. Susceptibility of *H*. *pylori* to this class of sulfonamides can be exploited by developing AAZ and similar compounds into novel anti-infective agents using the structural insights provided by this study.
