# Correction: Structural Basis for the Inhibition of *Helicobacter pylori* α-Carbonic Anhydrase by Sulfonamides

**DOI:** 10.1371/journal.pone.0132763

**Published:** 2015-07-10

**Authors:** The PLOS ONE Staff

The first author’s name is spelled incorrectly. The correct name is: Joyanta K. Modak. The correct citation is: Modak JK, Liu YC, Machuca MA, Supuran CT, Roujeinikova A (2015) Structural Basis for the Inhibition of Helicobacter pylori α-Carbonic Anhydrase by Sulfonamides. PLoS ONE 10(5): e0127149. doi:10.1371/journal.pone.0127149. The publisher apologizes for this error.
